# Enablers and Barriers to Community Engagement in Public Health Emergency Preparedness: A Literature Review

**DOI:** 10.1007/s10900-017-0415-7

**Published:** 2017-08-24

**Authors:** Anna Ramsbottom, Eleanor O’Brien, Lucrezio Ciotti, Judit Takacs

**Affiliations:** 1 Bazian Ltd, London, UK; 2 University of Amsterdam, Amsterdam, The Netherlands; 3 European Centre for Disease Prevention and Control, Stockholm, Sweden; 4 Centre for Social Sciences at the Hungarian Academy of Sciences, Budapest, Hungary

**Keywords:** Public health policy, Emergency preparedness, Community, Institution, Barrier, Enabler

## Abstract

Public health emergency preparedness (PHEP) all too often focusses only on institutional capabilities, including their technical expertise and political influence, while overlooking community capabilities. However, the success of institutional emergency preparedness plans depends upon communities and institutions working together to ensure successful anticipation, response and recovery. Broader community engagement is therefore recommended worldwide. This literature review was carried out to identify enablers and barriers to community and institutional synergies in emergency preparedness. Searches were undertaken across bibliographic databases and grey literature sources. The literature identified was qualitative in nature. A qualitative, ‘best fit’ framework approach using a pre-existing framework was used to analyse the literature, whereby themes were added and changed as analysis progressed. A working definition of community was identified, based on a ‘whole community’ approach, inclusive of the whole multitude of stakeholders including community residents and emergency management staff. Given the diversity in community make-up, the types of emergencies that could be faced, the socio-economic, environmental and political range of communities, there are no set practices that will be effective for all communities. The most effective way of engaging communities in emergency preparedness is context-dependent and the review did draw out some important key messages for institutions to consider.

## Introduction

Countries worldwide are encouraged to have effective plans in place that consider and aim to mitigate the social and economic disruption of entire communities in the event of emergencies that will inevitably occur [1].

International policy acknowledges the importance of such plans. The Sendai Framework for Disaster Risk Reduction 2015–2030 was adopted in 2015 at the Third UN World Conference in Sendai, Japan [2]. It recommends broader community engagement in the development of international, national and local policy on risk management and emergency response. The International Health Regulations adopted in 2005 is an agreement between 196 countries including all World Health Organisation Member States to work together for global health security, by building capacities to detect, assess and report public health threats [3]. Decision 1082 was adopted in 2013 by the EU to improve the response to emergencies, protecting citizens from a wide range of threats, particularly future pandemics and cross-border threats to health [4].

However, it is unclear whether these international agreements and the global commitment to community and institutional preparedness through relationship building and engagement, necessarily translates into action. Typically, public health emergency (PHE) plans involve little consultation with the public and are instead top-down, guided heavily by government and public health agencies, along with scientific experts [5].

At present, there seems to be a gap between evidence and practice in terms of synergies between communities and institutions and how institutions can engage communities. This may adversely affect the ability of institutions and communities to be prepared and effectively respond to emergencies. In this context we define an institution as a formal body with public health functions, and we mean by community a socially and/or spatially defined group with particular shared characteristics (such as geographic location, cultural practices, beliefs etc.), where community membership can be based on self-identification and/or external attribution.

A literature review was commissioned by the European Centre for Disease Prevention and Control (ECDC) with the research question: what are enablers and barriers to communities and institutions working together in the context of emergency preparedness? Presented here are the key findings from that literature review.

## Methods

The scope of the literature review was expanded from public health emergency and disaster preparedness to include more community-related terms and synergy-related concepts such as ‘working together’, based on the results of initial searches. These searches indicated that these community-related terms were particularly important in the aim of investigating community and institutional synergies in emergency preparedness.

**Fig. 1:**
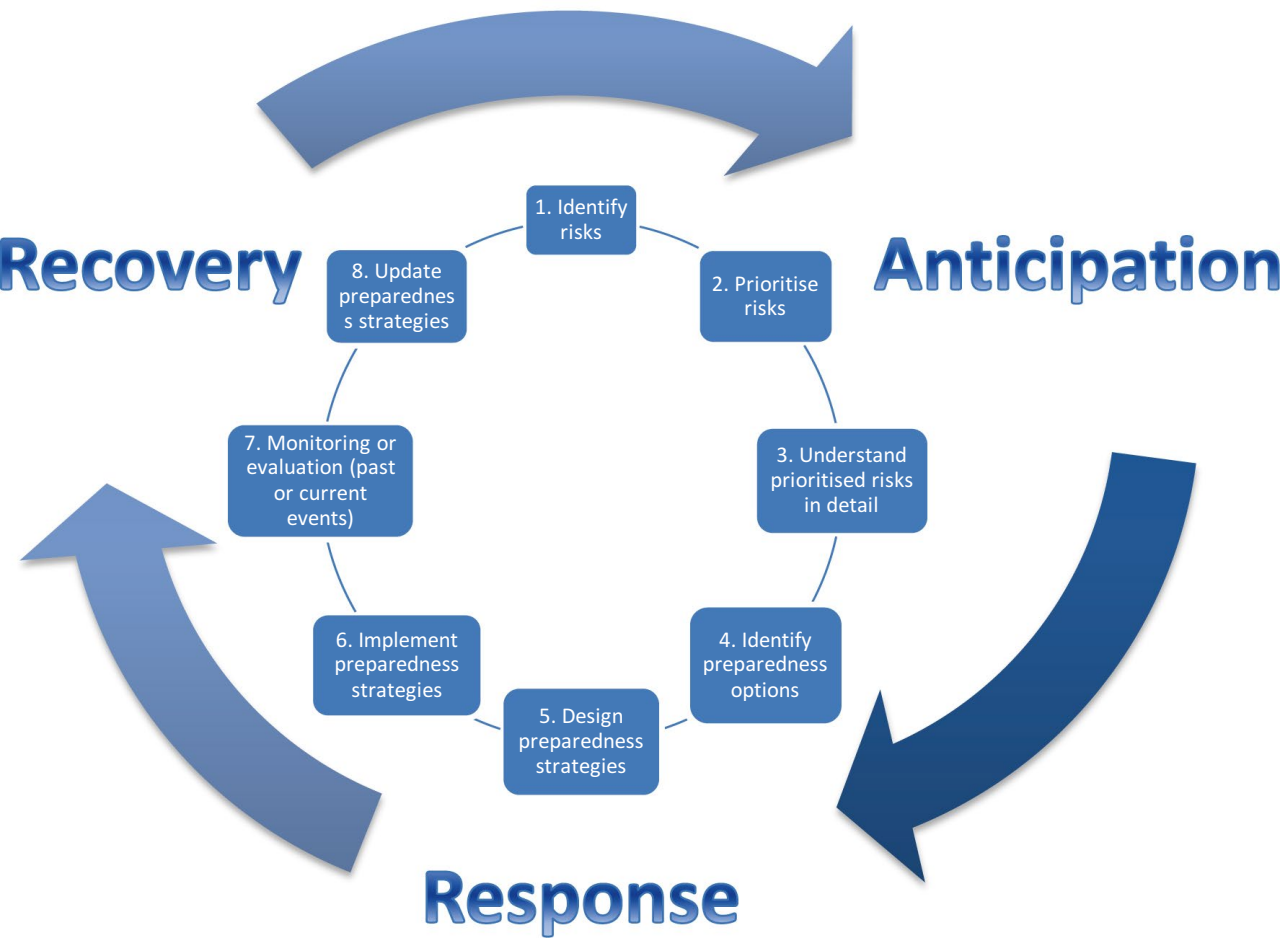
The preparedness cycle

Although communication of information, knowledge, advice and preparedness techniques will vary depending on the stage in the preparedness process, all stages of the preparedness cycle, anticipation, response and recovery (Fig. 1), are important in the success of overall preparedness. Therefore, literature was not limited to any specific stage in a preparedness cycle.

**Fig. 2:**
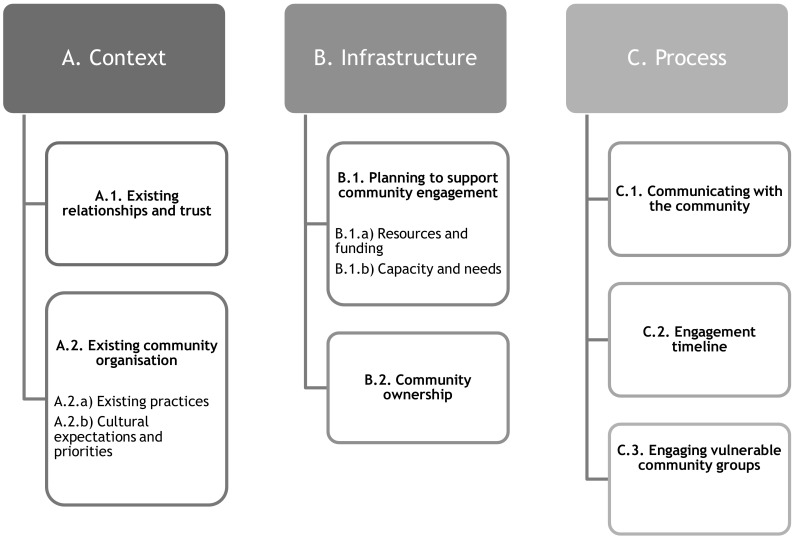
Adapted framework

An a priori assumption in developing the methodology was that it is axiomatic that public health institutions should aim to maintain a strong relationship with communities throughout the emergency preparedness cycle. According to a pre-existing framework, developed by the National Institute of Health and Care Excellence (NICE), ‘working together’ should be centred on three main themes: context, infrastructure and process (Fig. 2) [6]. This framework was adapted for this study; subthemes were added and modified to be representative of the identified literature.

### Searching

Search terms were identified using the citation pearl growing method, using the articles identified in the original scoping search [7]. Searches were performed across the interdisciplinary database Scopus (including MEDLINE) and grey literature sources (Google Advanced, Google Scholar, preventionweb.net and cdacnetwork.org) for examples of enablers and barriers to community and institution synergies [8, 9]. Consultation of experts was also carried out to identify additional references and searching the reference lists of included documents.

The search approach was designed to identify themes and to be both effective and efficient in capturing the most relevant literature.

### Sifting

Criteria for inclusion in the review were studies that: described engagement of communities in emergency preparedness; examined synergies between institutions and communities, looking at any phase of emergency preparedness: anticipation, response and/or recovery; were published between 2000 and 2016.

A global focus was taken, not excluding studies from any country. Studies were not excluded based on study type or language.

**Fig. 3:**
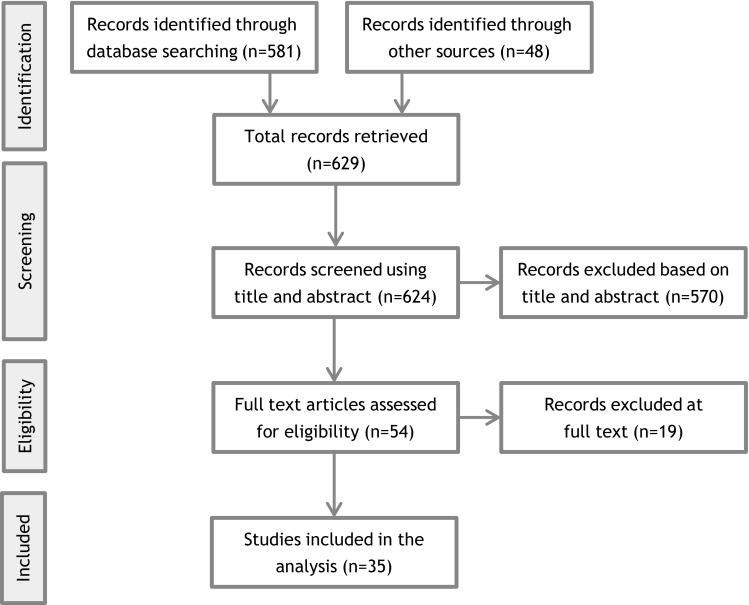
PRISMA diagram

The search and sift process is presented in a PRISMA diagram in Fig. 3. The searches are not fully exhaustive, though the three-pronged approach is designed to capture the most relevant literature.

### Analysis

A pre-existing framework was identified, focused on barriers and facilitators of community engagement in the public health domain, as a tool for analysing the data gathered in this study [10]. The qualitative framework analysis used a ‘best fit’ approach, using the framework as a foundation [11]. As the analysis progressed, new themes were added and existing themes were modified.

## Results

In total, 35 documents were included describing factors influencing community and institution synergies in emergency preparedness [1, 5, 12–44]. On the basis of the examined literature a working definition of ‘community’ was identified that was referenced in multiple articles and deemed most relevant to PHE preparedness [44]. The whole community approach was developed in response to the increasing frequency and effects of both natural and manmade emergencies: the concept defines a community as a dynamic entity that changes and adapts with variations in environmental, social and political factors [5]. Having a definition of community that is fluid and adaptable to various circumstances had the benefits of allowing an investigation of a broader variety of barriers and enablers, some which would not have been regarded if utilizing a more rigid definition.

Most of the examined literature focussed on the anticipation phase of the PHE preparedness cycle. Context, infrastructure and process are mainly associated with this phase as it is where the majority of planning takes place. The key issue for institutions is whether this translates into better prepared communities and institutions when responding to an emergency situation and future recovery.

### Context

This theme is mostly relevant for the anticipation and planning stage of the PHE preparedness cycle. Background information about communities and how well they work with institutions in anticipating an emergency will determine how successful the efforts are later on in the response and recovery phases of the emergency preparedness cycle.

A supportive, collaborative relationship between institutions and communities from the outset that is culturally sensitive and brings a wide range of organisations and people together will generate a more efficient response to emergencies [35].

#### Existing Relationships and Trust Between the Community and Institutions

A barrier may be a lack of community trust in government bodies that is rooted in perceived past injustices towards or continuing inequalities within the community [19, 35]. Trust is a prerequisite of community engagement and if community leaders lack trust in emergency management staff, they may decide to disengage with its representatives [36].

Strengthening relationships and trust in general between grass-roots communities and institutions can be assisted by creating a method for institutions to communicate their resilience priorities with the community [33].

#### Existing Community Organisation

##### Existing Practices

Another barrier to communities and institutions working together effectively is that certain groups may be left out of PHE emergency plans [43]. Local collective practices that are already in existence, such as where people seek information from and how decisions are made, need to be supported and stakeholders empowered, for example, by having open discussions of key roles and responsibilities [5].

One way to do this is to incorporate emergency planning discussions into community meetings that are already in place. Barriers to attending these meetings, such as lack of childcare or travel provision, must be addressed where possible to make sure those who want to play a role are able to [5].

However, emergency management staff should acknowledge that not all community conversations and decisions take place in these community-level forums. Therefore, it is important to delve deeper and find out where these discussions occur, such as places of worship or community centres [5].

##### Cultural Expectations and priorities

Initiatives for community engagement should acknowledge the embedded culture and value systems within communities. There may be higher priorities within a community, including immediate issues such as social- or healthcare, that need addressing before community members will consider engaging in emergency response [31, 36].

Initiatives for community engagement should also understand that people within a community sometimes have unreasonable expectations of some institutions. Community and institution synergies can be enabled by empowering communities to be less reliant on the government, for example by effectively using community leaders to reach the community they will become more self-sufficient and take ownership of the response and mitigation initiatives [29].

### Infrastructure

The infrastructure includes the resources and funding granted to and within a community. This will determine the extent to which community members can own community emergency response initiatives or would benefit from some assistance [5]. The infrastructure will be important to improve and strengthen during the anticipation phase so that during response and recovery, communities are able to take ownership, particularly at times when institutions are not able to provide support.

#### Planning to Support Community Preparedness

##### Resources and Funding

PHE preparedness agendas can become a burden for a community when not paired with an increase in resources [20, 39]. In order to enable successful synergies, funding should be made available for the population targeted for PHE preparedness improvement [19]. Flexibility in fund allocation can also allow innovative partnerships and programmes to be supported, where communities feel it most worthwhile [1, 31]. Increasing funding for diversity and cultural competency, such as translated materials and recruiting a diverse range of staff, is another important enabler [1, 14].

##### Capacity and Needs

Institutions do not always recognise the capacities of communities (such as networks of mutual support or trusted local channels of information etc.) from the outset. Therefore, community members may be reluctant to invest their resources in institution-facilitated community projects, preferring to invest their resources elsewhere [39].

Mapping already available capacities within communities can constitute an effective tool to support planning and prioritisation,[13] to identify which hazards may affect the whole community and those which may only affect certain groups of people vulnerable to the hazard [19]. By community mapping institutions can gain a richer understanding of community capacity, it can also increase awareness of community capacity amongst community members and demonstrate engagement, all of which contribute to improved PHE preparedness and response resilience [36].

#### Community Ownership

National government and public health institution-based initiatives rarely include the perspectives of the local community; they may not be able to stray too far from a centralised approach and community issues may be thought too culturally sensitive to consider in PHE preparedness strategies [39].

However, while decentralising resources can give communities independence,[15, 31] political support and experience is additionally needed to make decisions with the best outcomes [33]. This is a potential barrier to community independence and ownership from the outset.

A sense of ownership is a key component in ensuring effective community engagement in preparedness planning. In some PHE planning activities, communities should be leading—rather than following—in identifying priorities, organising support, initiating programmes and evaluating these initiatives. Communities that lead will have an incentive to make sure action and involvement are sustained [5, 31].

In practice, self-reliance often requires enhancing capacity through institutional support to enable local institutional and community access to- and control of funds that could be made available by national government systems [39]. To empower communities effectively, communities require training to develop and use local initiatives and opportunities [35]. This gives a sense of community ownership, by making plans that are tailored for communities and by communities [5].

### Process

Since communities are dynamic, complex entities, no uniform approach will fit every context. It is important to apply flexible approaches to community preparedness that are adaptable to a diversity of communities and environments [31]. The approach taken to PHE preparedness will be important in the anticipation, response and recovery stages as success in each of these is dependent on the action taken.

#### Communicating with the Community

A barrier to community-institution synergies is that communication may not be tailored to the demographics and cultural characteristics of groups within the community, essential to ensure the communication reaches the whole community. For example, the internet provides a powerful platform for non-governmental organisations (NGOs) and other institutions to gather and share information. However, this might not be an effective mode of communication for everyone, as some or all community members may not have the technical skills to access the information, and perhaps have no or limited internet access [35].

The communication of information, knowledge, advice and preparedness techniques will vary depending on the community context and infrastructure, as well as the stage in the preparedness cycle [45].

Successful synergies can be enabled by ensuring the information is coherent and consistent, so that information is perceived as reliable and therefore communities trust this information provision [35].

The communication process should be two-way and methods of communication should be made to enable feedback from all groups within the community so that all relevant local knowledge can be shared [24, 35].

#### Engagement Timeline

One barrier that may emerge throughout the engagement timeline is that communication methods that are effective during the anticipation phase are not effective in the response phase. During the anticipation phase, a lack of communication may result in poor preparedness and chaos when an emergency does occur, as community members are most likely first responders to an emergency [31]. During the response phase, effective communication should be the government’s responsibility, and their failure to live up to that may lead to community members being uninformed and potentially result in a state of panic [31].

To facilitate success at the start of the engagement process, as many people as possible need to be involved and low-level initiatives should take place such as mass media campaigns and information meetings [36].

As the process progresses, needs change and therefore the method of engagement should also change. Increasing levels of engagement will result in more consultation and two-way communication, with community members providing feedback. Fewer people are likely to be involved at this stage and ideally these will be representatives of the whole community and be able to report back to their networks [36].

#### Engaging Vulnerable Communities

Certain communities might be socio-economically disadvantaged or otherwise marginalised, and therefore not have the resources or financial ability to be prepared nor the coordinated knowledge of how to engage [23]. Here we describe only two of the many communities potentially vulnerable in a PHE emergency such as linguistically isolated populations and tourists.

Linguistically isolated communities may not be aware of the need to be prepared and may not have adequate plans in place for emergencies [23]. Word of mouth tends to be a more useful way of reaching these communities, as reported by them, with visual information as reinforcement. In these cases, friends are a very important source of information and knowledge: if they think it important to take action, others are likely to follow suit.

In order to engage effectively with communities, emergency preparedness staff should be educated on the diversity of the community and cultural competency exercises should be undertaken, such as building relationships with a multi-lingual community members to make outreach more effective [5].

Temporary visitors to an area provide another example of a community that needs to be considered since they will not have relationships that enable them to connect with preparedness plans and activities. In Iceland, where a volcanic eruption occurs every 3 to 4 years, tourists need to be aware of the warning systems and emergency responses to volcanic risk [25]. Tourists may be keen to receive information; however, a barrier is that they may not have this knowledge provided when visiting the area. To enable effective synergies, tourism professionals should receive special training in the early warning systems and how to disseminate information on emergency response procedures to tourists.

## Discussion

In our review, we applied the ‘whole community’ approach that included a working definition of ‘community’ we used throughout the analysis. We found that the ‘whole community’ approach was the most inclusive definition of community as it captures the full spectrum of individual community members as well as community organisations. It is a dynamic concept that changes with shifting environmental, socio-economic and political factors.

When considering the community context, existing relationships are important. Mistrust of institutions is a deep-seated barrier that can have historic roots and be difficult to change. By creating ways for institutions to communicate their emergency preparedness objectives and to reach out to community leaders, trust can be built and relationships formed, which can be used as levers for action.

Where possible, communities should tap into their internal resources when preparing for, responding to, and recovering from emergencies. However, communities may need assistance in locating resources, with which external institutions can support them.

In order to create sustainable community emergency preparedness, the community should take ownership of initiatives. This review suggests that institution-run actions may not incorporate the perspective of the community, so any decisions to start with should be, at the very least, made jointly.

Engaging vulnerable groups within the community was a theme that repeatedly emerged in the literature. There are many groups within a community that may be more vulnerable to emergencies for a variety of reasons. Finding appropriate ways to reach out and engage each of these communities is important to consider when preparedness initiatives are being developed.

This review acknowledges that the importance of factors are context-dependent and, to facilitate community engagement, offers the following key points for consideration:

- *Establishing relationships & building trust* Community and institution synergies should be meaningful; achieved by institutions listening to community priorities. This builds trust by ensuring communities feel they are being heard.
- *Mapping existing networks & planning resources* Enablers and barriers can be identified by community mapping, whereby all elements of a community are considered from the community members’ perspective and resource needs identified. This exercise is on-going and therefore context-specific, depending on the threat type a community must prepare for.
- *Developing cultural competencies* Members of institutions initiating community engagement should be culturally competent. They should be aware of how information will be received by groups and any translations and/or sign language material requirements.
- *Ensuring two-way communication & community ownership* In emergency preparedness, there is often one-way communication, from institutions to communities. Communication should be two-way, acknowledging the needs and capacities of communities.
- *Engaging throughout & considering vulnerable groups* Institutions should engage communities in the anticipation phase of the preparedness cycle, with special attention to vulnerable groups. They should maintain this relationship throughout the preparedness cycle.

### Strengths

Strengths are our inclusive definition of community, the inclusion of all steps of the emergency preparedness cycle (even though not all literature used the same framework), the inclusion of studies from a variety of contexts and various types of institutions. The adaptation of the public health barriers and enablers framework also ensured the themes were relevant to the literature identified, and indicated enablers and barriers specific to the community engagement in emergency preparedness.

### Limitations

There may be enablers and barriers that have not been studied or written about in the literature.

While some of the enablers and barriers identified are relevant to multiple contexts, some may be context-dependent. There are certain countries where there is a plethora of research with a specific political, socio-economic and environmental landscape and therefore those findings may not be applicable to other countries. There were fewer examples from a European context and from the field of communicable diseases.

### Research Recommendations

Future publications relating to community and institution synergies in emergency preparedness should have greater focus on the relationship between all phases of the PHE preparedness cycle and whether PHE preparedness initiatives in the anticipation phase lead to a more effective response and recovery. There is also need for more studies focussing on communicable diseases in this context.

Although some vulnerable groups were identified, there are many more that may not have been researched. The groups described in this article were indicative of those included in the literature, rather than exhaustive. Future preparedness initiatives should take this into account.

## Conclusion

The identification of enablers and barriers in institutional and community preparedness in terms of their working together adds to the evidence base in this area and helps decision-makers identify how to engage communities more effectively. The adapted framework is a useful starting point for institutions wishing to engage communities in emergency preparedness. The end goal is to minimise adverse outcomes in emergency situations through building a partnership between communities and institutions in iterative processes, adapting to changing contexts.
